# Radiomics approach for identifying radiation-induced normal tissue toxicity in the lung

**DOI:** 10.1038/s41598-024-75993-y

**Published:** 2024-10-16

**Authors:** Olivia G. G. Drayson, Pierre Montay-Gruel, Charles L. Limoli

**Affiliations:** 1 Department of Radiation Oncology, University of California, Irvine, CA 92697-2695 USA; 2 Antwerp Research in Radiation Oncology (AReRO), Centre for Oncological Research (CORE), University of Antwerp, Antwerp, Belgium; 3 Dept. of Radiation Oncology, University of California, Irvine, CA 92617-2695 USA

**Keywords:** Radiomics, Radiotherapy, Extracellular vesicles, Machine learning, Cancer, Computational biology and bioinformatics, Medical research, Oncology

## Abstract

The rapidly evolving field of radiomics has shown that radiomic features are able to capture characteristics of both tumor and normal tissue that can be used to make accurate and clinically relevant predictions. In the present study we sought to determine if radiomic features can characterize the adverse effects caused by normal tissue injury as well as identify if human embryonic stem cell (hESC) derived extracellular vesicle (EV) treatment can resolve certain adverse complications. A cohort of 72 mice (*n* = 12 per treatment group) were exposed to X-ray radiation to the whole lung (3 × 8 Gy) or to the apex of the right lung (3 × 12 Gy), immediately followed by retro-orbital injection of EVs. Cone-Beam Computed Tomography images were acquired before and 2 weeks after treatment. In total, 851 radiomic features were extracted from the whole lungs and < 20 features were selected to train and validate a series of random forest classification models trained to predict radiation status, EV status and treatment group. It was found that all three classification models achieved significantly high prediction accuracies on a validation subset of the dataset (AUCs of 0.91, 0.86 and 0.80 respectively). In the locally irradiated lung, a significant difference between irradiated and unirradiated groups as well as an EV sparing effect were observed in several radiomic features that were not seen in the unirradiated lung (including wavelet-LLH Kurtosis, wavelet HLL Large Area High Gray Level Emphasis, and Gray Level Non-Uniformity). Additionally, a radiation difference was not observed in a secondary comparison cohort, but there was no impact of imaging machine parameters on the radiomic signature of unirradiated mice. Our data demonstrate that radiomics has the potential to identify radiation-induced lung injury and could be applied to predict therapeutic efficacy at early timepoints.

**Supplementary Information:**

The online version contains supplementary material available at 10.1038/s41598-024-75993-y.

## Introduction

Radiomics is an emerging field that involves the extraction and analysis of quantitative data from medical images^1,2^. The technique was developed to extract more information on tumor tissue that supplements clinical data and can better predict tumor progression^3,4^). These data include types of features such as intensity, shape, and texture which can provide information about tissue characteristics and the biological behavior of tumors^2–5^. In conjunction with machine learning, radiomic data can be used to improve diagnosis, prognosis, and treatment planning for cancer patients^5,6^. It can also help to identify biomarkers that can predict response to therapy and guide personalized treatment^7^. Combined with other “omic” data in what is now called “multiomics”, radiomics is an essential part of modern personalized oncology therapy and treatment planning.

In the field of oncology, radiomics is of particular interest in assessing the efficacy of radiotherapy and chemotherapy treatments^8,9^). Radiomics can be applied to assist in staging of the disease, identification of genetic features, discrimination of healthy and unhealthy tissue for treatment planning and treatment monitoring through prediction of remission, treatment outcome or survival^5^. While radiomics is a tool that originated from and is predominantly utilized in the field of oncology, it is starting to be expanded to other tissue types and pathologies including liver fibrosis^10^, impaired pulmonary function^11^, functional magnetic resonance imaging^12^ and radiation induced injury^4,8^. The strong contrast of the lungs in Computed Tomography (CT) images makes it a popular organ of interest in testing the applications of radiomics. A series of investigations have utilized radiomics to distinguish between COVID-19 pneumonia and pneumonia with differing etiologies^13–16^. In one such study^14^ the radiomic features outperformed radiologists’ assessment in predicting COVID-19 pneumonia severity and patient outcome. Radiomics is starting to be investigated in the context of radiation induced toxicity with promising prognostic results^17^.

Radiation-induced lung injury (RILI) remains a prominent cause for morbidity in patients receiving thoracic radiotherapy and is the dose-limiting toxicity for management of esophageal cancer^8,18^ and lymphoma^8,19^. RILI is dependent on several factors including dose and irradiated volume, therefore methods of accurately predicting adverse outcomes as well as techniques to mitigate injury are urgently needed^20,21^. Preclinical studies are an essential avenue to look for biomarkers that can be used in clinical studies to identify sensitive tissue and inform treatment plans. It has been shown that classifiers trained on a single texture feature achieve good accuracies at predicting radiation pneumonitis (inflammatory response ~ 2 weeks post-irradiation) in both low-dose and high-dose regions of the lungs^22^. In another study, both individual and multiple-feature classifiers were capable of identifying radiation pneumonitis and a correlation between change in feature value and radiation dose was found in 20 radiomic features^23^. Our current study focused on applying these techniques on mice after hypofractionated thoracic radiation therapy to determine if radiomic features can be used to distinguish between irradiated and unirradiated animals.

Extra-cellular vesicles (EV) are membrane-bound vesicles produced by most cells for intercellular communication and contain a wealth of bioactive cargo such as proteins, lipids, mRNA, and microRNA^24,25^. While they can be mediators of disease, attention is turning to utilize them as therapeutic interventions in the central nervous system^26^, as well as other organ sites^27,28^. Recent work from our laboratory has shown that stem-cell derived EV treatment can mediate neuroprotection similarly to stem cell therapy while avoiding the risks of tumorigenesis^29,30^. In a previous study, we demonstrated that retro-orbital injection of stem-cell-derived EVs into mice was able to improve survival and mitigate lung inflammation (pulmonary pneumonitis) induced by a single dose of X-ray irradiation^31^. Therefore, the secondary aim of this study was to expand upon the efficacy of EV treatment by evaluating two hypofractionated irradiation paradigms: a whole-lung irradiated cohort and a locally irradiated cohort. In addition to a Cone-Beam Computed Tomography (CBCT) image analysis, radiomic features were also evaluated to determine whether EV mitigation of radiation-induced pneumonitis can be detected by quantitative imaging analysis. Given the significant impact radiation-induced pneumonitis has on patient health and quality of life, this method demonstrates the potential for detecting and mitigating lung injury and therefore improving cancer treatment outcomes.

## Results

### Experimenter-dependent 2D lung density measurements fail at identifying radiation-induced lung injury

**Fig. 1:**
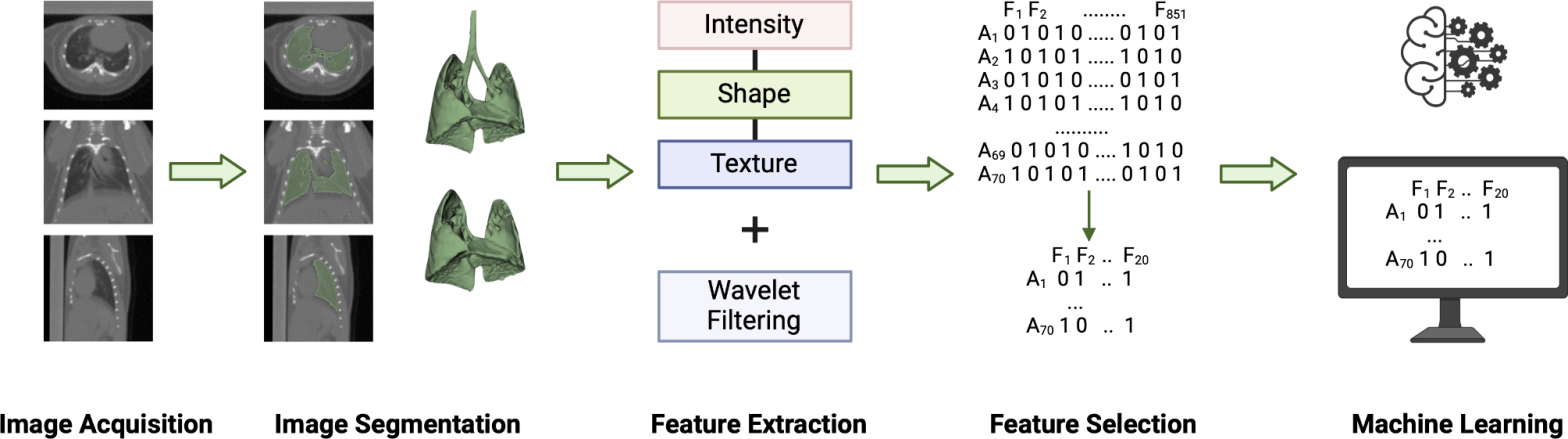
Radiomic analysis workflow for this study. Image segmentation and feature extraction were conducted in 3DSlicer. Feature selection and machine learning analysis were performed in R. No image pre-processing was conducted on this dataset prior to feature extraction.

**Fig. 2:**
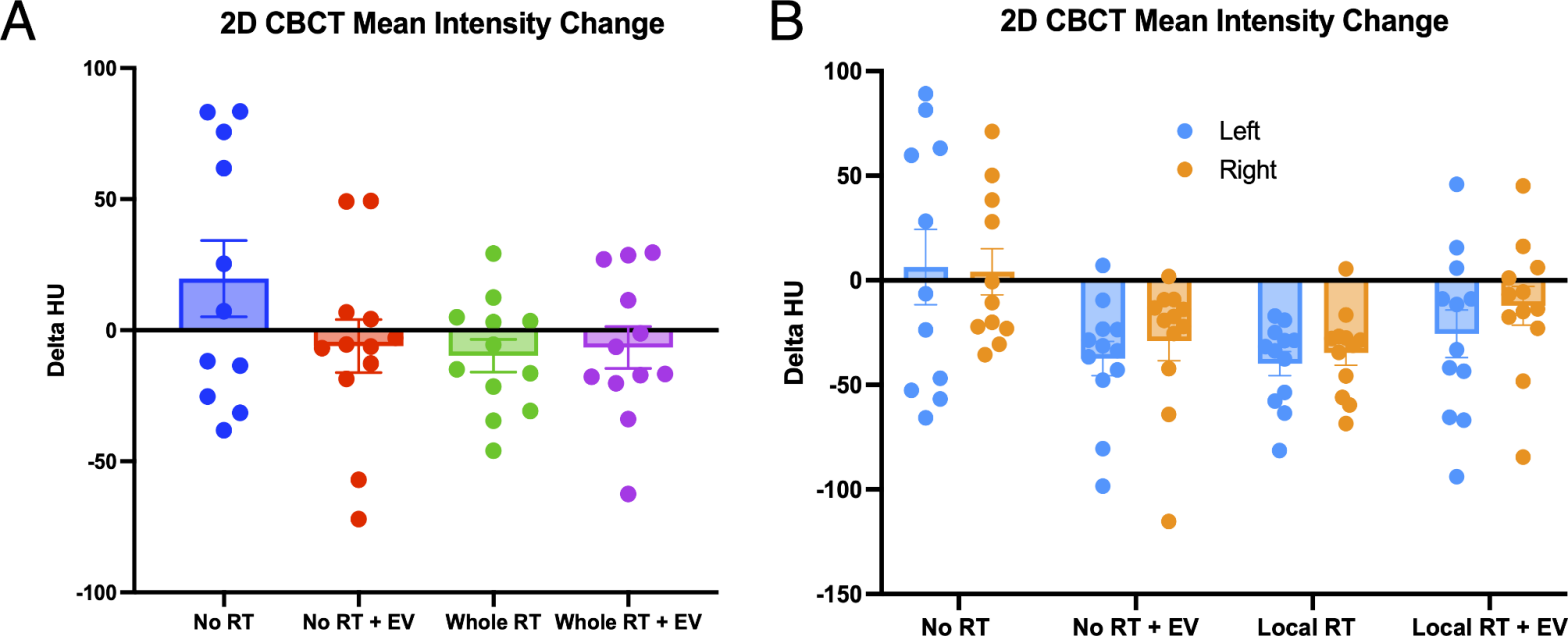
Change in Mean Intensity of CBCT lung images from a single 2D slice in Osirix Lite software. (**A**) The change in mean intensity measured for the whole lung 2 weeks after radiotherapy (RT) and/or extracellular vesicle (EV) injection. (**B**) The change in mean intensity measured separately for the left and right lungs in the locally irradiated cohort.

A schematic of our experimental timeline is shown in Fig. 1. With the aim of identifying radiation induced lung inflammation and lung injury, lung density was measured by CBCT at 2 weeks post-exposure. Our previous results on animals irradiated with a single dose of 14.4 Gy showed a significant increase in lung density in the lung of irradiated animals 2 weeks post-exposure, measured after 2D manual contouring^7^. This increase in lung density was not observed in the animals treated with a single injection of hESC-derived EV. In the present study, at a similar time point and using the same 2D quantification method, no differences in HU were observed in any of the treatment groups (see Fig. 2), suggesting an absence of radiation-induced lung injury at early timepoint after the delivery of 3 × 8 Gy total lung or 3 × 12 Gy to the apex of the right-lung. All CBCT data was tested for normality (whole lung *p* = 0.0601, local irradiation: left lungs *p* = 0.007, right lungs *p* = 0.0776) and then either parametric or non-parametric ANOVA tests. In the whole lung irradiated cohort normality was shown (*p* = 0.0601) and ANOVA test showed no significant differences between groups (F(3,43) = 1.789, *p* = 0.1636). In the locally irradiated lungs the mean intensity was calculated for each lung separately and the distributions were not consistently Gaussian (left lung: *p* = 0.007, right lung: *p* = 0.0776). The Kruskal-Wallis test showed global significance (*p* = 0.0158, Kruskal-Wallis statistic = 17.26) but the multiple comparisons tests were all non-significant. No protective effect of hESC-derived EV could be confirmed with our experimental design. For this reason, and to rule out the possibility that the absence of difference in lung density could be due to the 2D quantification technique, we performed the radiomic analysis on the same set of data.

### Principal component analysis indicates a distinction between irradiated groups and controls

**Fig. 3:**
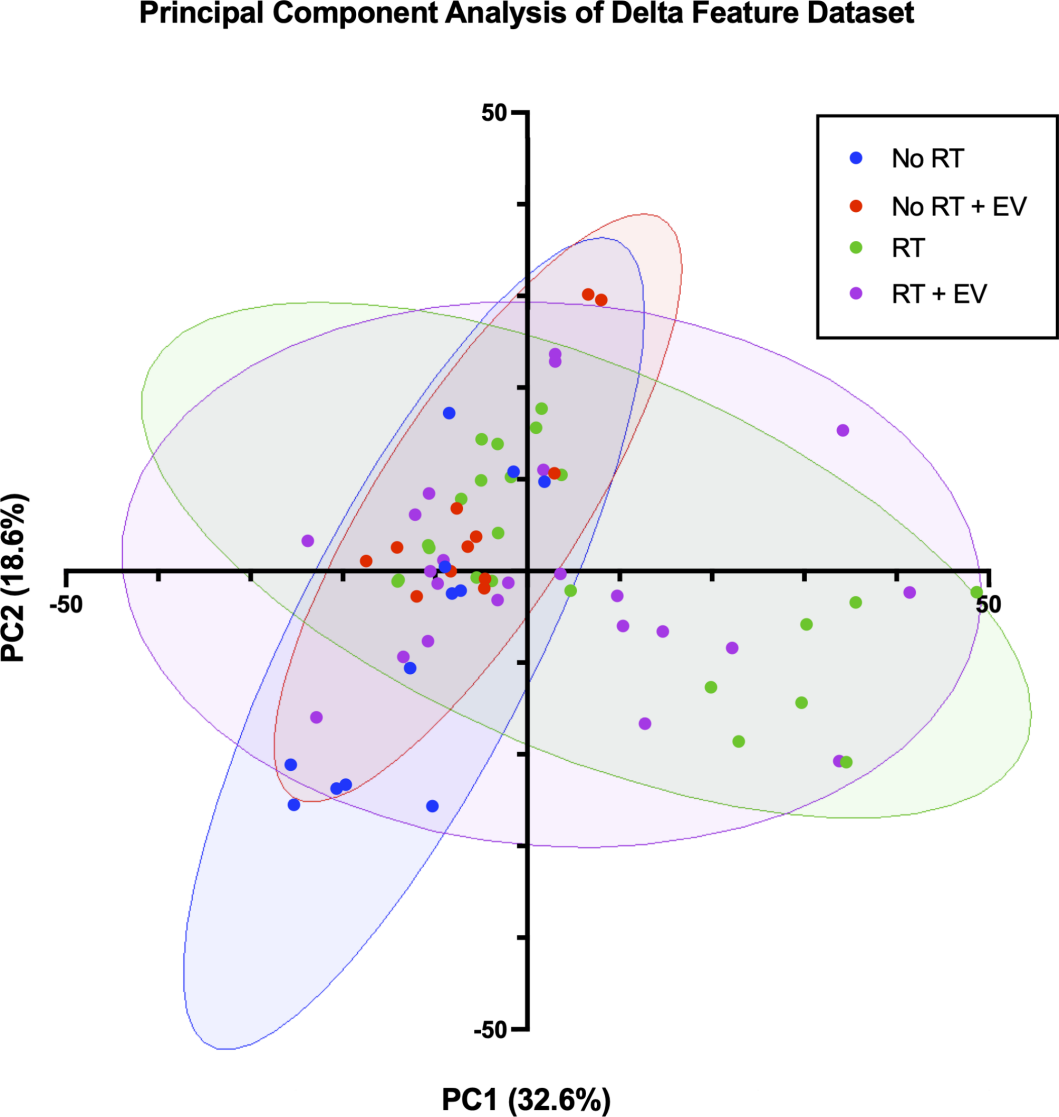
Scatter plot of the two principal components with the greatest contribution to the variance in the dataset. Each datapoints represents the net feature change of an animal from baseline to week 2. Treatment status was hidden during PCA and is shown by the ellipses (blue is the unirradiated and vehicle injected control group, red is the unirradiated and EV injected group, green is the irradiated and vehicle injected group, and purple is the irradiated and EV injected group).

A Principal Component Analysis (PCA) produced 70 principal components with the two most representative of the spread of the data comprising 32.6% (PC1) and 18.6% (PC2) of the variance respectively. The net change features from each animal were plotted against PC1 and PC2 as shown in Fig. 3. The figure indicates overlap of the control and unirradiated EV groups as well as overlap between the irradiated vehicle and the irradiated EV groups. However, a large proportion of the irradiated animals both with and without EV treatment, are distinct from the unirradiated groups. A significant difference between the PC clusters was observed between the control and irradiated clusters (*p* = 0.0073 from PC1) and a trend was observed between the control and the irradiated with EV treatment clusters (*p* = 0.0523 from PC1). This suggests that a radiation effect is observed which is not significantly mitigated by the injection of EVs. The plot suggests that a classifier could be trained to distinguish irradiated animals from controls with high accuracy.

### Machine learning models are capable of distinguishing irradiated, and EV treated animals from controls

Three supervised classification models were trained and tested on this dataset for a prediction task to determine if an animal had received a given treatment based on fewer than 20 radiomic features. Two feature selection algorithms - Feature Importance (FI) and Recursive Feature Elimination (RFE) – were compared and optimized for classification accuracy (see Tables 1 and 2). RFE was the higher performing method and used to select the feature set for all 3 classifiers. The selected features are shown in Table 3. The radiation and EV classifiers were both binary classification models which utilized the full dataset but only trained on predicting one treatment type. The multi-class classification model (the treatment classifier) was trained to predict all 4 classes of treatment. As expected, the features selected for the treatment classifier overlapped with features selected for the two binary classifiers. However, no features were selected for all 3 models.

**Table 1:** Performance of radiation classifier with varying feature selection methods, feature set size and train/test split.

Feature selection method	Size of feature set	Predictor variable	Classification model	Train/test split	AUC	Accuracy (%)	Confidence intervals	*P*-value (* if < 0.05)	Sensitivity	Specificity
Recursive feature elimination	16	Radiation	Random forest	50/50	0.909	94.3	80.8–99.4	* 2.62 × 10^−4^	0.818	1.00
Feature importance	16	Radiation	Random forest	50/50	0.909	94.3	80.8–99.4	* 2.62 × 10^−4^	0.818	1.00
Principal components	16	Radiation	Random forest	50/50	0.534	57.1	39.4–72.7	9.46 × 10^−1^	0.182	0.750
Recursive feature elimination	16	Radiation	Random forest	60/40	0.913	92.9	76.5–99.1	* 5.89 × 10^−3^	0.875	0.950
Recursive feature elimination	16	Radiation	Random forest	70/30	0.917	95.2	76.2–99.9	* 8.03 × 10^−3^	0.833	1.00
Recursive feature elimination	16	Radiation	Random forest	80/20	0.955	92.9	66.1–99.8	1.65 × 10^−1^	1.00	0.909
Recursive feature elimination	13	Radiation	Random forest	50/50	0.770	82.9	66.35–93.44	* 2.09 × 10^−2^	0.583	0.957
Recursive feature elimination	23	Radiation	Random forest	50/50	0.750	80.0	63.06–91.56	* 1.02 × 10^−2^	0.500	1.00

**Table 2:** Performance of EV classifier with varying feature selection methods, feature set size and train/test split.

Feature selection method	Size of feature set	Predictor variable	Classification model	Train/test split	AUC	Accuracy (%)	Confidence intervals	*P*-value (*if < 0.05)	Sensitivity	Specificity
Recursive feature elimination	14	EV	Random forest	50/50	0.859	85.7	69.7–95.2	* 2.28 × 10^−5^	0.941	0.778
Feature importance	14	EV	Random forest	50/50	0.832	82.9	66.4–93.4	* 1.13 × 10^−3^	0.941	0.722
Principal components	14	EV	Random forest	50/50	0.546	54.3	36.7–71.2	4.34 × 10^−1^	0.647	0.444
Recursive feature elimination	14	EV	Random forest	60/40	0.795	78.6	59.1–91.7	5.7 × 10^−2^	0.923	0.667
Recursive feature elimination	14	EV	Random forest	70/30	0.727	71.4	47.8–88.7	6.15 × 10^−2^	1.00	0.455
Recursive feature elimination	14	EV	Random forest	80/20	0.750	71.4	41.9–91.6	2.11 × 10^−1^	1.00	0.500
Recursive feature elimination	21	EV	Random forest	50/50	0.801	80.0	63.1–91.6	* 1.4 × 10^−3^	0.812	0.790
Recursive feature elimination	34	EV	Random forest	50/50	63.1	60.0	42.1–76.1	5.73 × 10^−1^	0.476	0.786

**Table 3:** Selected features determined by recursive feature elimination and utilized for the training and test datasets for the supervised classifiers. The wavelet prefix is included in the feature names.

Radiation Classifier	Treatment group classifier	EV status classifier
Original minor axis length		HHH small area low gray level emphasis
HLH run entropy		HLL Idmn
LLH dependence entropy		HLH first order Kurtosis
LHH MCC		HHH first order skewness
LLH sum entropy		HLH first order minimum
HLL MCC		HHL size zone non-uniformity normalized
Original elongation	HHL zone entropy	
LLH Imc2	HLL first order Maximum	
LLL Contrast	LLH run entropy	
Original maximum 2D diameter row	LHH first order mean	LLH Imc1
LHL gray level non-uniformity	Original Max2D diameter slice	LHH small dependence low gray level emphasis
LLH gray level non-uniformity normalized		HHL small area low gray level emphasis
HLL correlation		HHH joint entropy
HLL small area high gray level emphasis		HHH Imc2
LLH first order skewness		
LLL small dependence emphasis		

**Fig. 4:**
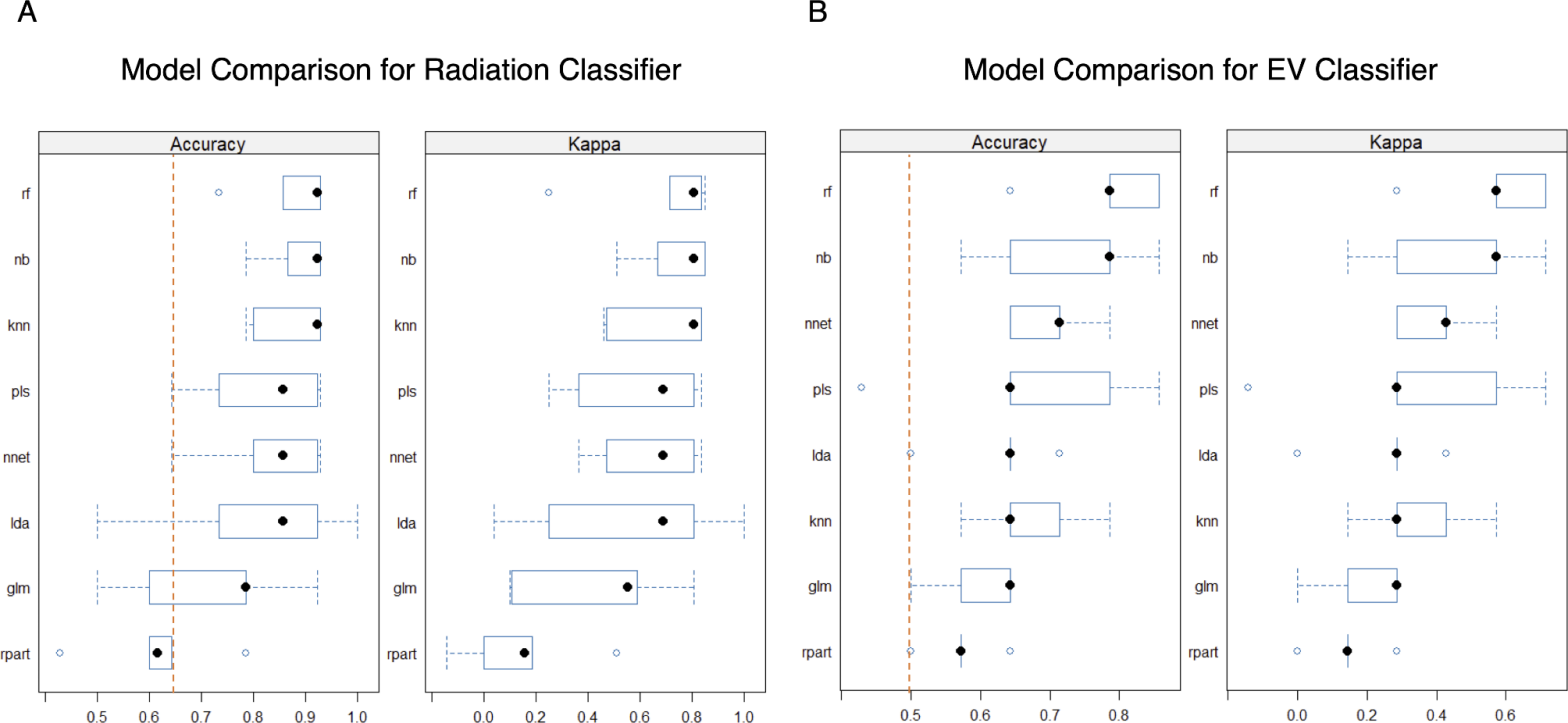
Box and whisker plots comparing accuracy and kappa score of several classification models for the Radiation and EV classifiers. Dotted red line indicates the no-information rate. rf – Random Forest, nb – Naïve Bayes, knn – k-Nearest Neighbors, pls – Partial Least Squares, nn – Neural Net, lda – Linear Discriminant Analysis, glm - Generalized Linear Model, rpart - Classification and Regression Trees (CART).

For both binary classifiers, the impacts of changing the feature selection method, classification model, and hyperparameters were assessed. Of the 8 models evaluated (Random Forest, Naïve Bayes, k-Nearest Neighbors, Partial Least Squares, Neural Net, Linear Discriminant Analysis, Generalized Linear Model, Classification and Regression Trees (CART)), the random forest model was the most accurate and had the highest Kappa score for both the radiation and the EV classifier (see Fig. 4). These models were selected for comparison due to the variety in methods including decision trees, neural network, linear classification models etc. On this basis and also due to its prominent use in the literature^32^, the random forest classification method was chosen.

Using the same classification model parameters, feature selection methods were also compared including feature importance, recursive feature elimination and principal components with feature sets of varying size (see Tables 1 and 2). Recursive feature elimination with the 16 or 14 selected features was consistently better performing and therefore was used. In addition, other train/test splits and feature set were evaluated (see Tables 1 and 2) to select the hyperparameters.

**Fig. 5:**
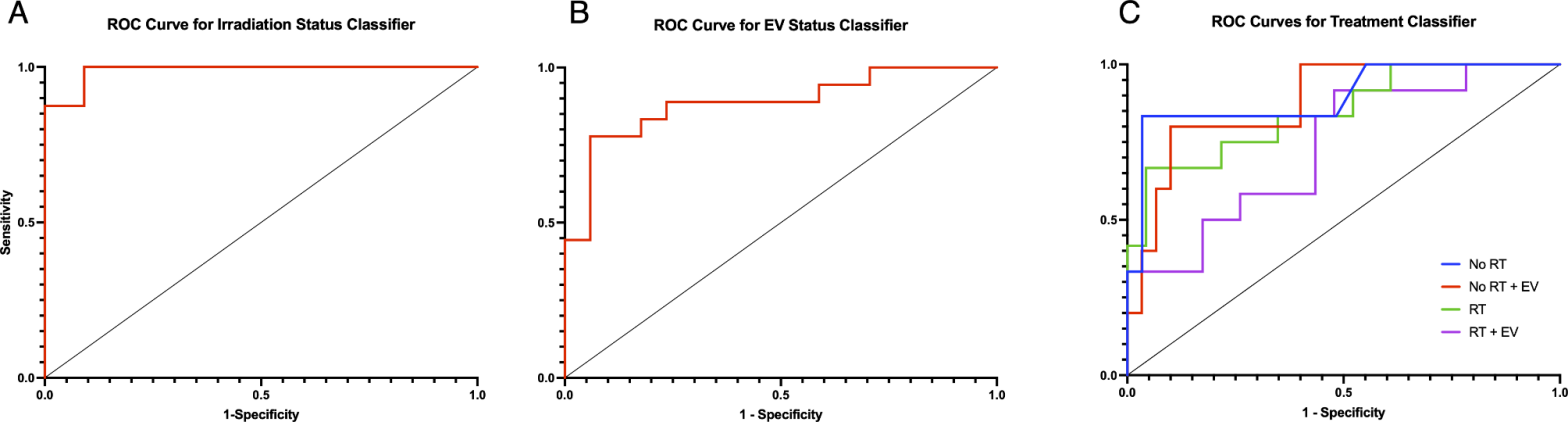
Receiver Operating Characteristic (ROC) Curves for the three classifiers. (**A**) Results of binary radiation classifier trained on features shown in first column of Table 3. (**B**) Results of binary EV classifier trained on features shown in third column of Table 3. (**C**) Results of multiclass classifier trained on features shown in second column of Table 3.

All the optimized classification models were able to achieve good accuracy, statistically significantly greater than the no information rate. The highest performing classifier was the radiation classifier which achieved an accuracy of 94.29% and an area under the curve of 0.909. The ROC curves and metrics are shown in Fig. 5; Table 4. The EV classifier achieved an accuracy of 85.71% and an AUC of 0.859. The treatment classifier attained an accuracy of 65.71% with all binary distinctions achieving an accuracy greater than the no information rate. The two classes that the treatment classifier was most accurate at distinguishing was between the control group and the radiation plus EV injection group. The worst performing distinction was between the radiated plus vehicle group and the radiation plus EV injection group. These results suggest therefore that a radiation effect is more evident than an EV effect alone and that no mitigation of the radiation effect from the EV treatment was observed.

**Table 4:** Metrics of the 3 random forest classification models, each trained on the same subset of animals with the features shown in Table 3. The metrics are calculated from the test subset.

Classifier	Radiation status	EV status	Treatment
Accuracy (%)	94.29	85.71	57.14
AUC/mean AUC	0.909	0.859	0.796
Sensitivity (%)/mean sensitivity	0.818	0.941	0.529
Specificity (%)/mean specificity	1.00	0.778	0.842
No information rate (%) (p-value)	68.57 (2.6 × 10^−4^)	51.43 (2.3 × 10^−5^)	34.29 (4.7 × 10^−2^)

### Significant radiation effect observed in 3 radiomic features but no EV effect

**Fig. 6:**
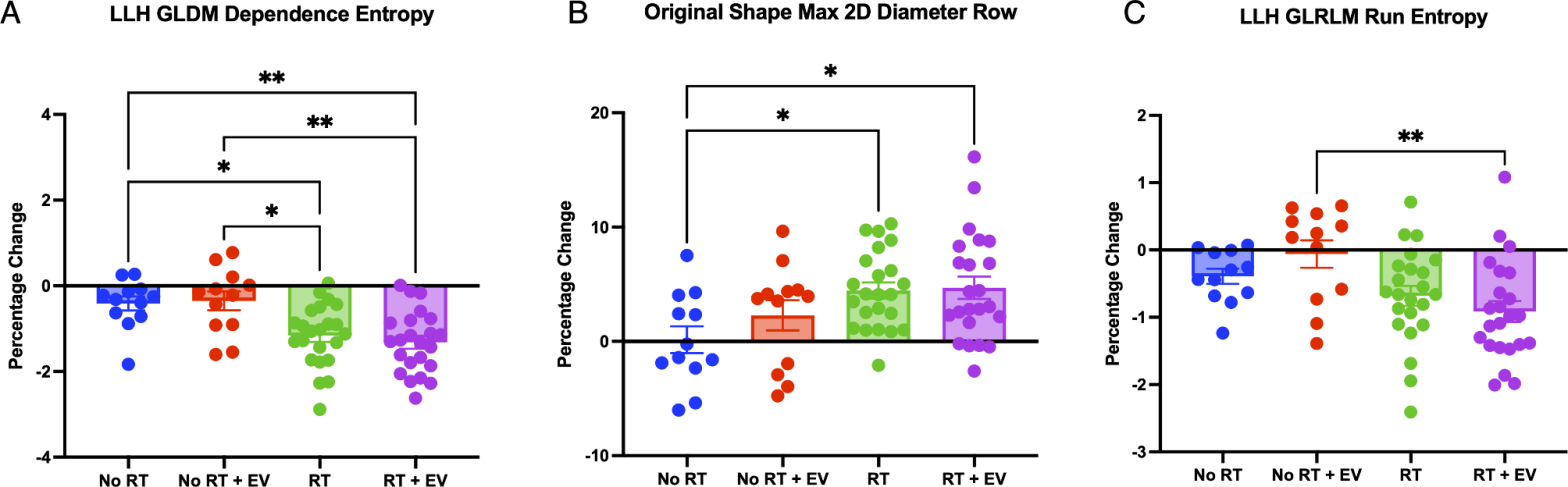
Bar graphs of the radiomic features which showed significant differences between treatment groups from one-way ANOVA and t-tests. (**A**) Plot of LLH filtered Gray Level Dependence Matrix Dependence Entropy (**B**) Plot of unfiltered image shape feature Maximum 2D Diameter Row (**C**) Plot of LLH filtered Gray Level Run Length Matrix Run Entropy.

Of the radiomic features selected by RFE, three features were found to have significant differences between treatment groups using One-Way ANOVA and Bonferroni multiple comparisons tests. These features (as shown in Fig. 6) are GLDM Dependence Entropy from LLH wavelet image (F(3,66) = 8.019, *p* = 0.0001), Max 2D Diameter Row from the unfiltered image (F(3,66) = 3.959, *p* = 0.0117), and GLRLM Run Entropy also from the LLH wavelet image (F(3,66) = 4.639, *p* = 0.0053). Normality was confirmed in all three features (Dependence Entropy: *p* = 0.6798, Max 2D Diameter Row: *p* = 0.9792, Run Entropy: *p* = 0.7149). A difference between an unirradiated group and an irradiated group was observed in all 3 features but no difference between vehicle-injected and EV groups was observed, nor an EV sparing effect, in support of the indication by the PCA.

### Radiomic features identify changes in the locally irradiated lung

**Fig. 7:**
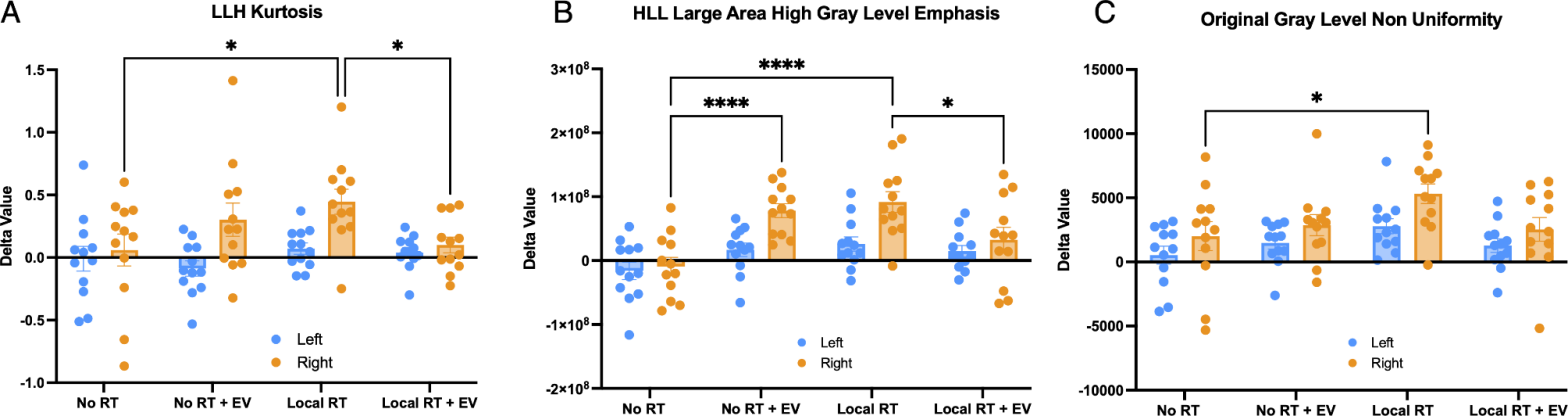
Bar graphs of a set of radiomic features which demonstrated a significant EV effect not statistically different from controls at 2 weeks in the locally irradiated animals. Blue indicates the unirradiated left lung and Orange indicates the irradiated right lung. (**A**) Plot of LLH Intensity feature Kurtosis (**B**) Plot of HLL filtered Gray Level Run Length Matrix feature Large Area High Gray Level Emphasis (**C**) Plot of unfiltered Gray Level Size Zone Matrix feature Gray Level Non-Uniformity.

In the locally irradiated cohort, RFE was repeated, and the selected features inspected. The left and right lungs were split during image segmentation and a set of radiomic features were extracted for each lung individually. It was found that three radiomic features showed significant differences between groups as assessed by ANOVA (Fig. 7). These features were the intensity feature Kurtosis from the LLH wavelet image, the GLRLM Large Area High Gray Level Emphasis feature from the HLL wavelet image, and the Gray Level Non-Uniformity feature from the unfiltered image. The observed differences were only in the right lung and were predominantly between the unirradiated vehicle injected control lungs and the irradiated vehicle injected lungs. However, one feature (Large Area High Gray Level Emphasis) showed a surprising significant difference between the unirradiated vehicle group and the unirradiated EV treated group. In addition, no differences between the unirradiated vehicle injected control lungs and the irradiated EV treated lungs were seen, suggesting an EV sparing effect. Normality was verified for each feature and each lung individually (kurtosis: left *p* = 0.0663, right *p* = 0.1080; emphasis: left *p* = 0.5337, right = 0.5140; non-uniformity: left *p* = 0.0513, right *p* = 0.1593).

### Radiomic features are not impacted significantly by CT imaging machine or imaging parameters

**Fig. 8:**
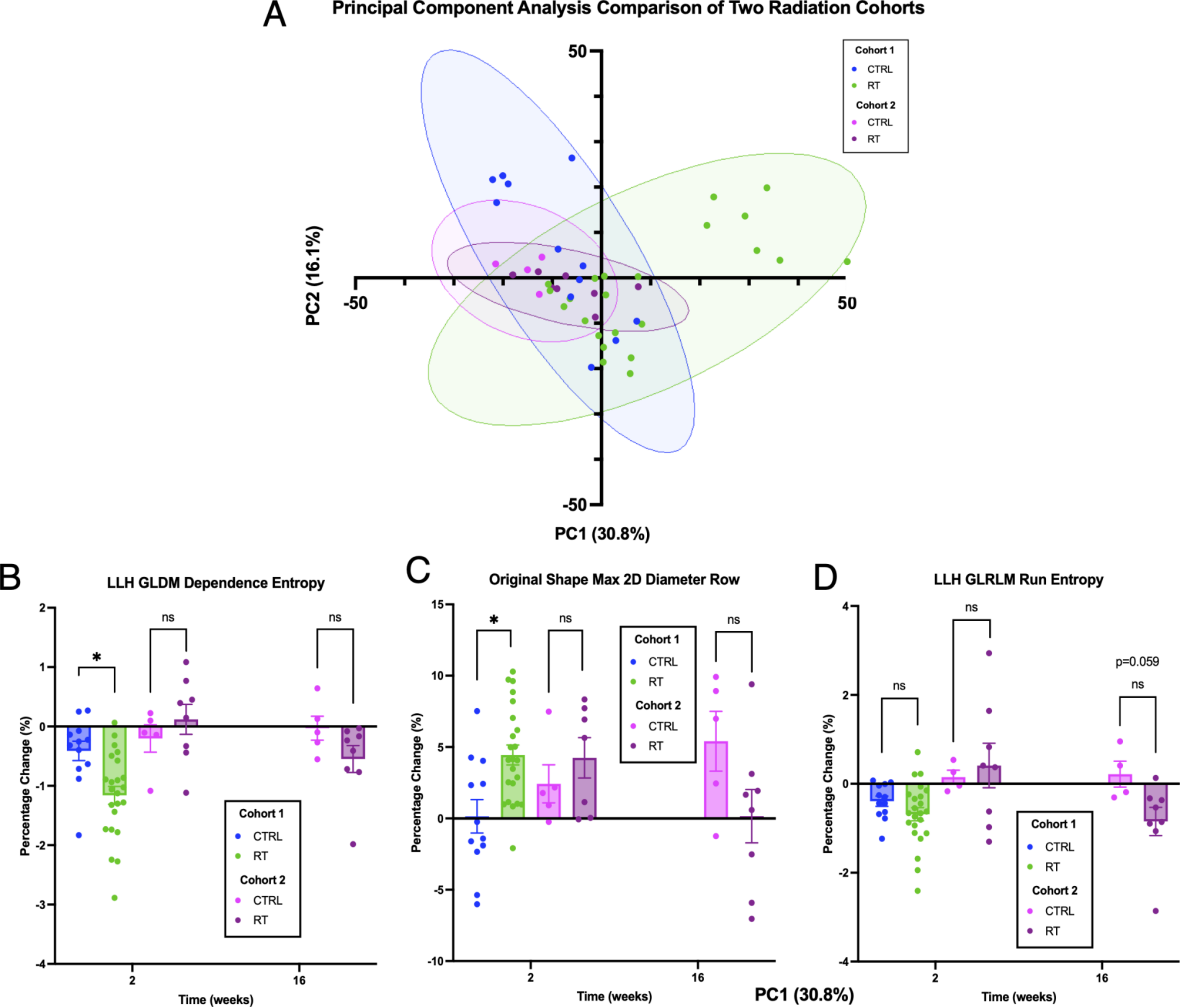
Comparison of the radiomic signature of the original cohort with a small independent cohort. (**A**) PCA of the original fractionated cohort without EV injection (from Fig. 3) and the comparison cohort. (B-D) Bar graphs of the features plotted in Fig. 6 including both the 2-week and 16-week timepoints of the comparison cohort. (**A**) Plot of LLH Intensity feature Kurtosis (**B**) Plot of HLL filtered Gray Level Run Length Matrix feature Large Area High Gray Level Emphasis (**C**) Plot of unfiltered Gray Level Size Zone Matrix feature Gray Level Non-Uniformity.

A small cohort of unirradiated controls (*n* = 5) and thoracically irradiated mice who received one fraction of 14 Gy (*n* = 4) or 15 Gy (*n* = 4) were compared to the main cohort. A PCA was conducted on both cohorts and the results plotted in Fig. 8A. No deviation from the controls of the original cohort was seen in either the controls or the irradiated animals from the secondary cohort (t-test of PC1 between controls from each cohort: *p* = 0.216 and between groups within cohort 2: *p* = 0.07). This suggests that one fraction of 14–15 Gy was not sufficient to produce the deviation seen in a subset of the 3 × 8 Gy cohort. However, it also demonstrates that the controls from both cohorts, while imaged on different machines in different countries, years and imaging parameters, do not have significantly different radiomic signatures. The three radiomic features selected above (Fig. 8B-D) were also plotted for the secondary cohort at the 2-week and 16-week timepoint. While no statistically significant difference was observed between the controls and the irradiated mice at the 2-week timepoint, there was again no difference between the controls from the primary cohort and the controls from the secondary cohort, supporting the PCA indication that the radiomic signature of both the controls and the irradiated lungs from the smaller secondary cohort were not distinct from the original controls. However, at 16 weeks the trend did return in the wavelet-LLH dependence entropy and run entropy with a slight decrease in the run entropy of the irradiated images though not significant (*p* = 0.059).

## Discussion

While deep learning shows increasing promise in classifying medical images and extracting features from huge datasets, the opacity of the decision-making being done by these algorithms hinders the translation into medical applications. It is especially the case for diagnosis and prognosis, where the logic at each step of the decision process is vital for the medical professional and patient to understand. Therefore, much attention is being drawn to the field of radiomics, where features are engineered and therefore can be replicated between institutions in efforts to both streamline workflow and derive a unified and comprehensible meaning. In addition, using traditional machine learning models such as decision tree classifiers or logistic regression to analyze these features instead of neural networks, allows for transparency throughout the image analysis process. These radiomic features are still being studied thoroughly to ensure they are robust, stable over time, and independent of the device used, image modality, imaging parameters, and other potential confounders.

Studies of cancer patients receiving radiotherapy have found success in predicting radiation pneumonitis using radiomic features in combination with clinical and dosimetric data^23,33–35^. Outside of the lung, radiomics has also been employed in several human studies to improve prediction of radiation-induced toxicity including in osteoradionecrosis^36^, acute xerostomia^37^ and rectal toxicity^38^. However, human cancer studies are limited by the confounding impacts of the cancer itself as well as concurrent treatments. This is where preclinical studies can better correlate radiomic features with specific stages of radiation-induced disease. Several murine studies have investigated radiomics in pulmonary diseases^39^ including interstitial lung disease^40^ compared with bleomycin-induced pulmonary fibrosis^40,41^, and air pollution induced lung disease^42^. One study published this year studied radiation pneumonitis and pulmonary fibrosis using deep learning radiomics with similar predictive capability^43^.

Despite 10 years of radiomics research, there remains an unmet need for predictive biomarkers of radiation-related morbidity^8^. Therefore, this study aimed to utilize radiomics in a manner that has not been investigated extensively to date, by using an animal model to determine if radiation toxicity could be distinguished using radiomic features.

The present results show no change in mean lung density measured in 2D slices from CBCT scans taken 2 weeks after exposure to hypofractionated doses of 3 × 8 Gy to the whole lungs and 3 × 12 Gy to the apex of the right lung, along with no impact from EV injection. However, from the 3D radiomic analysis, principal component analysis suggested a radiation effect but no EV effect which was supported by several radiomic features selected by Recursive Feature Elimination (RFE). These features were used to train and test a series of random forest machine learning classifiers which were able to predict treatment group with significant accuracy. The features selected by RFE for training and testing included predominantly texture and intensity features with a small number of spatial features. Radiation-induced changes in the shape feature Maximum 2D Diameter Row and texture features Dependence Entropy and Run Entropy were observed. The maximum 2D diameter row is the measure of the maximum width of the lung and therefore is an indication of increased lung volume in the sagittal plane^44^. This could be due to the larger expansion of the lungs during breathing in order to compensate for regions with damaged or inflamed tissue. Dependence and Run Entropy are indicators of the heterogeneity of texture patterns where the Dependence Matrix describes the dependency of connected voxels on the center voxel and the Run Length Matrix describes the distribution of runs of voxels of the same gray level. The fact these are both decreased in the LLH filtered images of the irradiated cohorts suggests the radiation-affected tissue is less heterogeneous than the healthy tissue in the wavelet image. The data showed that the inter-group differences were not solely intensity changes or spatial changes and that a variety of features were needed to characterize a particular phenotype.

A similar approach was conducted for a cohort of locally irradiated animals, except the left and right lungs were segmented individually before radiomic feature extraction. Statistical tests of the radiomic features indicated a radiation effect in the locally irradiated right lung that was not seen in the unirradiated left lung. Importantly, this approach uncovered a sparing effect of the EVs in the irradiated lung. The effect was found in features extracted from both the wavelet filtered images and the original image and was seen in both intensity and texture-based features. Kurtosis, or the degree of “tailedness” of a distribution was increased in only the irradiated right lung, indicating that more voxels have intensities near the mean. The Large Area High Gray Level Emphasis and Gray Level Non-Uniformity features are from the Size Zone and Run Length Matrices respectively and therefore both describe differences in the regions of connected voxels either in a zone or in a run. The data showed that the wavelet filtered images were able to amplify subtle patterns that may correspond with pathological endpoints.

A comparison with another set of CBCT images acquired from thoracically irradiated mice showed that the radiomic signatures of the control mice was unaffected by the CT machine or imaging parameters. However, the irradiated images did not show the same deviation in radiomic signature that was observed from the primary cohort. This is likely due to the much smaller sample size and differences in dose regimen, since a trend approaching significance appeared in one feature (wavelet-LLH Run Entropy) but only at 16 weeks post-irradiation.

The hurdle that is inevitably hit when analyzing large datasets is the risk of overfitting. By extracting > 800 features from a single image, it can be relatively easy to find features that fit any pattern the investigators choose. Therefore, care must be taken to minimize this risk as much as possible. This study was limited predominantly by the lack of accompanying histological data and the small sample sizes. The risk of overfitting was minimized by randomly splitting the dataset into independent training and testing subsets of equal size. In addition, all preclinical data including animal IDs were removed from the dataset during model training and testing to prevent data leakage. In order to verify the correlation of these radiomic features with radiation-induced changes, further studies are needed using validated histologic endpoints (e.g. lung fibrosis, collagen deposition and alveolar thickness etc.) along with a variety of imaging parameters and radiation doses, types, dose-rates, fractionation schedules etc.

In conclusion, this study is one of few to investigate if radiomic features are impacted by radiation-induced toxicity in the lungs of mice and shows the potential for radiomic features to support and identify subtle effects normally not captured through traditional metrics.

## Methods

### Animals

Animal experiments were approved by the Institutional Animal Care and Use Committee (IACUC) of the University of California Irvine and performed within institutional guidelines. 72 female C57BL/6J mice were purchased from Charles River Laboratories and housed at the UCI vivarium from 10 weeks of age. The mice were kept in standard conditions with access to rodent chow and water *ad libitum*. All mice were included in the study and were randomly divided into 6 treatment groups and received treatment at 8 weeks of age. All image analysis was performed by investigators blinded to the treatment groups. The study is reported in accordance with the ARRIVE guidelines^45^.

### Irradiation

The cohort (total of 72 mice, *n* = 12/group) consisted of 6 treatment groups. All animals were anesthetized with 2% isoflurane for both irradiations and injections including the unirradiated controls. The irradiated animals either received whole thorax irradiation delivered in 3 fractions of 8 Gy, or local thoracic irradiation delivered to the apex of the right lung in 3 fractions of 12 Gy. In both cohorts, fractions were spaced out by 48 h as has been done routinely to avoid consecutive irradiation and anesthesia days. The right apex of the lung was chosen to minimize the dose delivered to the heart. Irradiation was delivered using a SmART + X-ray cabinet (Precision Inc.) at 225 kV, 13 mA, with a 0.3 mm copper filter and delivered with one (local apex exposure) or two (whole lung) opposite vertical beams after fluoroscan imaging to position the mice at the treatment isocenter. The prescribed doses were determined at 10 mm depth with a 15 mm circular (whole lung) or 5 mm circular (local apex exposure) collimated fields according to previous depth dose and dosimetric measurements in solid water phantoms with calibrated EBT3 Gafchromic films. Alignment and positioning were confirmed before irradiation with lasers and fluoroscan imaging, with the collimator in place, using the rib cage, collar bone and diaphragm as anatomical landmarks to ensure correct positioning.

### Extracellular vesicles

Human stem cell derived EV were extracted using biweekly ultracentrifugation and filtration as described previously in^31^. Three retro-orbital injections of either 10^10^ hESC-derived EV or vehicle were performed immediately following each irradiation fraction or sham-irradiation while the mice were anesthetized. More details on the EV extraction and human stem cell culturing were already reported in )^51^.

### Cone-Beam Computed Tomography (CBCT) imaging

Lung density was monitored for each animal using CBCT also with the Precision SmART + small animal irradiator (80 kV; 1 mA, 2 mm aluminum filter, image spacing 0.1 mm), under isoflurane anesthesia. For each imaging day, a region of interest containing the entire thorax determined from a scout image on the first mouse and then would be used as the imaging boundary for the rest of the cohort. Images were acquired at baseline (the day of the first irradiation) and 2 weeks following the last irradiation fraction.

### 2D and 3D lung segmentation

Lung contouring and reconstruction were performed using the Osirix Lite Software (version 14, https://www.osirix-viewer.com/) on a single section of the lung. The image section containing the bifurcation of the right bronchiole was selected to maintain consistency across the cohort. The draw tool was used to manually contour the entire lung parenchyma and lung density was evaluated for each animal and each time point by the mean intensity in Hounsfield Units (HU). In the locally irradiated cohort, the left and right lungs were contoured separately and the mean intensity for each lung was measured. Values of ΔHU were calculated for each animal and time point by the formula:

ΔHU_t_ = HU_t_ - HU_0_ where t is the time after treatment and HU_0_ is the baseline value.

The stages of the radiomic analysis (see Fig. 1) were designed based on the traditional radiomic workflow as described in^1,46^. Whole lungs were segmented semi-automatically using the software 3DSlicer (version 4.11, https://www.slicer.org/). A 3D mask was created with a range of -900 Hounsfield Units (HU) to -200 HU for all scans. This range includes functional lung tissue (-700 to -600 HU) and tissue with inflammation or fibrosis (-600 to -200 HU) while excluding air (-1000 HU) and surrounding soft tissue (0-300)^47,48^. Therefore, the threshold of -900 to -200 HU was selected in order to keep both healthy and inflamed lung parenchyma within the segmented volume while removing all surrounding soft tissue. After thresholding, the trachea, bronchi, pulmonary vessels and any artefacts of the segmentation were manually removed using the scissor tool in 3DSlicer. In order to divide the left and right lungs for the local irradiation classification model, the lungs were manually cut in 3D Slicer also using the scissor tool after segmentation of the whole lungs as described above. The results of the 3D generated mean intensity are shown in Supplementary Material.

### Radiomic feature extraction

Radiomic features were calculated from the 3D mask using the 3D Slicer pyradiomics extension (version 3.0.1, https://pyradiomics.readthedocs.io, python version 3.6^49^). All the feature classes were extracted (Grey Length Dependence Matrix (GLDM), Shape based (2D and 3D), Grey Level Co-occurrence Matrix (GLCM), First Order Statistics, Grey Level Run Length Matrix (GLRLM), Grey Level Size Zone Matrix (GLSZM), and Neighbouring Grey Tone Difference Matrix (NGTDM)). The wavelet filter class was also applied to all scans to yield 8 derived images using the PyWavelets package (version 1.1.1, https://pywavelets.readthedocs.io). Voxels were not resampled and so maintained the pixel spacing of the image (0.1 mm), symmetrical GLCM was enforced and the bin width was fixed at the default value 25 as this would generate 28 bins which is within the recommended range of 16–128^49,50^). This method generated 851 features for each scan which were combined into a single data frame and analyzed in R Studio (version 4.1.2, https://posit.co/downloads).

### Feature selection

The extracted feature set contained data from image scans of the entire cohort (including whole thorax irradiated and locally irradiated) taken at baseline and two weeks after all treatment. Net change features were calculated for each animal by subtracting f(0), the value at baseline, from f(1), the value at two weeks (in a similar way to the calculation of delta HU features described above). Therefore, the feature set contained a row of 851 features for each animal in the cohort (*n* = 72). The feature set was reduced first through removal of low variance features (nearZeroVar function with a frequency cut of 95/5 and a unique cut of 10) and removal of highly correlated features (greater than 95% correlation). This cleaned dataset contained 268 features and was used for the Principal Component Analysis (PCA) and feature selection methods described below.

Three methods of feature selection were performed on the whole lung cohort. Firstly, PCA (prcomp function in RStudio, version 4.1.2) which allowed data visualization and dimensionality reduction. Data were centered and scaled, the Singular Value Decomposition (SVD) method was used to compute the principal components and ellipses were plotted with the stat_ellipse function assuming a normal distribution with a confidence interval of 95%. Secondly, feature importance (caret package, version 6.0–93) was measured using the treatment status as the predictor, then the highest ranking 20 features were selected. The third method of feature selection was Recursive Feature Elimination (RFE), (caret package, version 6.0–93) with a subset size of 20 features, random forest functions, and ten-fold cross-validation. These values were selected after fine tuning of both the RFE and supervised classifiers. RFE was chosen as the method of feature selection that performed best for classifier training and so was repeated for each predictor variable to create the feature set for the supervised machine learning models. In the evaluation of the locally irradiated cohort, RFE was the only method of feature selection performed. The selected features were inspected, and a statistical analysis was performed to determine if group differences were present.

### Machine learning analysis

Three classification models were trained and tested from the dataset, two binary classifiers aiming to predict radiation group or EV treatment group, and a multi-class classifier aiming to predict both radiation and EV treatment. Given the small size of the dataset, a 50:50 split was selected to divide the training set and test set. This way the chance of overfitting can be minimized while maximizing accuracy for both the training and test sets. Several train/test splits, feature selection models and classification models were evaluated and compared but the random forest model was ultimately chosen as it was consistently the most accurate. This is also supported in the literature on radiomics of lung CTs^32^. The random forest function ‘rf’ within the caret package (ranger) was used for all classifiers.

### Comparison cohort

A set of CBCT images from a smaller secondary cohort (*n* = 13 total) was used to compare radiomic features across different imaging devices and parameters. Female C57BL6 mice were imaged at baseline, 2 weeks and 16 weeks after receiving one fraction of 14 Gy (*n* = 4) or 15 Gy (*n* = 4) of thoracic X-ray irradiation (225 kV, 12 mA, cylindrical collimator with diameter 15 mm). Unirradiated controls (*n* = 5) were also imaged (40 kV, 3 mA) at the same timepoints. The irradiations and images were acquired on a SmART + machine located at the CHUV in Lausanne, Switzerland. The lungs were segmented in the same method described above and the same set of radiomic features was extracted. A PCA was conducted from the 2-week delta features from both cohorts and radiomic features were statistically compared that showed significant radiation effect in the fractionated cohort.

Assuming an alpha/beta ratio for the lung of 3 Gy as a late responding tissue, the Biologically Effective Dose (BED) of the first cohort is 88 Gy for the whole lung (3$\:\times\:$8Gy) and 180 Gy for the locally irradiated cohort (3$\:\times\:$12Gy). In the comparison cohort, the BEDs are 79.33 Gy (1$\:\times\:$14Gy) and 90 Gy (1$\:\times\:$15Gy). This shows that the comparison cohort received a similar BED to the whole lung irradiated cohort.

### Statistical analysis

Statistical analyses were carried out using GraphPad Prism (version 9, https://www.graphpad.com/features) software. CBCT data were first evaluated for normal distribution using the Shapiro-Wilk test and then analyzed using one-way ANOVA followed by Bonferroni multiple comparison test. If the data were found to not fit a Gaussian distribution, then the non-parametric Kruskal-Wallis ANOVA was conducted followed by the Dunn’s multiple comparison test. Data in the text are presented as means ± SEM, and all analyses considered a value of *P* ≤ 0.05 to be statistically significant.

## Electronic supplementary material

Below is the link to the electronic supplementary material.

Supplementary Material 1

## Data Availability

The data that support the findings of this study are available from the corresponding author upon reasonable request.
