# Supplementary material for: Radiomics approach for identifying radiation-induced normal tissue toxicity in the lung
Source: Sci Rep. 2024 Oct 16;14:24256. doi: 10.1038/s41598-024-75993-y (PMC11484882; doi:10.1038/s41598-024-75993-y)
Supplement: Supplementary file 1 — Supplementary Material 1 [file 41598_2024_75993_MOESM1_ESM.docx]

**Supplementary Material**

**Histological Staining of Lung Inflammation**

Half of the cohort was euthanized by cervical dislocation. Lungs were sampled and gently inflated by the injection of 1 ml of FineFIX (#84-1717-00/Biosystems) directly in the trachea. The organs were then fixed in the same solution and kept at 4°C before being paraffin embedded and cut into 4 µm sections. The sections were stained with a solution of hematoxylin-eosin (HE) and Sirius Red, examined using an inverted brightfield microscope (Evos XL Core/Thermo Fisher Scientific). Histological analyses of the images did not reveal significant staining indicative of inflammation or fibrotic tissue. Representative images from each treatment group are shown in Supplementary Figure 1.

**
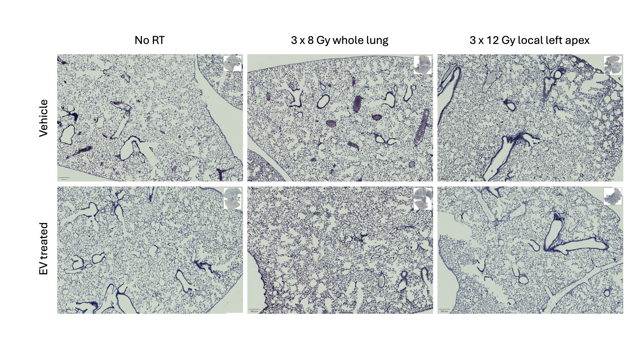
Supplementary Fig. 1**

**Supp. Fig. 1**: Control and irradiated lungs were stained for histology (H&E and Sirius Red). Representative images do not show signs of inflammation or fibrosis across the treatment groups analyzed.

**3D and 2D Segmentation Methods**

To ensure the reliability of the 2D mean intensity calculation, the radiomic output of the mean intensity across the entire lung was collected and plotted in Supplementary Figures 2 and 3. As the figures show, the trends observed (though non-significant) were reproduced in the whole lung calculation except for a significant difference between the No RT group and the No RT + EV in the whole lung irradiated cohort 3D measurement. This outlines the efficacy of the slice selection and 2D estimation method and that first order intensity measures are not always capable of describing changes in lung CBCT images after irradiation, especially when used as a stand-alone measurement.

**Supplementary Fig. 2**


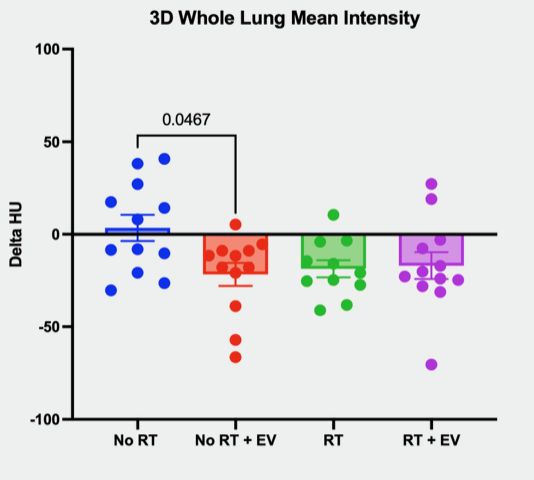


**Supp. Fig. 2**: Radiomic output of the mean intensity was collected and plotted for the whole lung for the cohorts shown. While there was a significant drop in density between the first two cohorts (No RT vs No RT+EV), density changes across the entire lung for the three treatment groups was not evident.

s9uwere stained for histology (H&E and Sirius red). Representative images do not show signs of inflammation or fibrosis across the treatment groups analyzed.

**Supplementary Fig. 3**


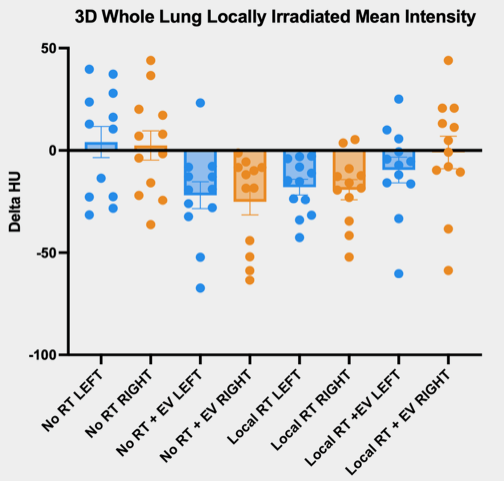


**Supp. Fig. 3**: Radiomic output of the locally irradiated mean intensity was collected and plotted for the whole lung for the cohorts shown. While the trends in changes of density were reproduced in the 3D calculations, they did not reveal significant changes across the treatment groups.
